# Association between coffee and caffeine consumption and chronic kidney disease

**DOI:** 10.1038/s41598-025-11543-4

**Published:** 2025-07-12

**Authors:** Panpan Gao, Xinrong Ji, Weiwei Wang, Yao Chen, Zhan Gao, Zengli Yu

**Affiliations:** 1 The Third Hospital affiliated Zhengzhou University, Zhengzhou, China; 2 The Fifth Hospital affiliated Zhengzhou University, Zhengzhou, China; 3 College of Public Health, Zhengzhou University, Zhengzhou, China; 4 NHC Key Laboratory of Birth Defects Prevention, Changsha, China

**Keywords:** Coffee, Tea, Caffeine, Chronic kidney disease, NHANES, Kidney diseases, Chronic kidney disease

## Abstract

Chronic kidney disease (CKD) is a significant global health issue associated with cardiovascular complications. While coffee, tea, and caffeine consumption have been linked to various health benefits, their relationship with CKD remains unclear. We conducted a cross-sectional analysis of NHANES 1999–2018 data, including 49,827 participants, assessing dietary intake through two 24-hour recalls and determining CKD status via the CKD-EPI equation. Our results showed that after adjusting for covariates, higher coffee consumption (> 352.5 g/day) was associated with lower odds of CKD (OR 0.760, 95% CI 0.701–0.823), as was tea consumption (OR 0.913, 95% CI 0.843–0.89). The highest tertile of caffeine consumption also showed a significantly reduced odds of CKD (OR 0.734, 95% CI 0.674–0.799). These findings suggest that coffee, tea, and caffeine consumption may be associated with better renal function, highlighting potential dietary strategies for CKD prevention.

**Supplementary Information:**

The online version contains supplementary material available at 10.1038/s41598-025-11543-4.

## Introduction

Chronic kidney disease (CKD) is a growing public health problem that significantly impacts patients’ quality of life and leads to higher rates of illness and premature death^1–3^. From 1990 to 2017, the global prevalence and mortality of CKD increased by 29.3% and 41.5%, respectively^3^. CKD can lead to renal dysfunction and progress to end-stage kidney disease and cardiovascular disease (CVD)^4^. In addition, CKD related complications can lead to the acceleration of disease progression and the increased risk of cardiovascular-related morbidity^4^. Multiple factors (i.e., alcohol use, smoking, and obesity) were found to be associated with the risk of CKD^5,6^. Recent research has increasingly focused on the relationship between dietary patterns and CKD risk^7^. As a crucial dietary component, the consumption of beverages is intricately intertwined with the preservation of overall health and renal function, as well as the modulation of high-risk factors for CKD progression (e.g., hypertension, obesity, and diabetes)^7–9^.

The association between coffee consumption and multiple health outcomes (i.e., stroke, hypertension, and CVD) has been extensively studied^10,11^. Multitude epidemiologic studies indicate that coffee may protect against various health outcomes, including hypertension, diabetes mellitus, and CVD^10,11^. The association between coffee consumption and CKD risk has been widely studied, while results are inconsistent. Previous studies reported that daily coffee intake was associated with a decreased risk of the development of CKD^12,13^. A cohort study found that coffee consumption was positively associated with CKD risk, but the association was not significant^14^. A meta-analysis suggested that coffee consumption might present potential harmful effects on kidney health^15^. Caffeine, a prominent bioactive compound found in coffee, has been reported to be associated with an increase in the estimated glomerular filtration rate (eGFR) and a reduced risk of developing CKD^16^. However, contrasting findings have also been reported where increased caffeine consumption did not significantly alter eGFR^17^.

Epidemiologic studies have also examined the association of tea consumption with CKD risk and the eGFR^18–20^. The majority of the studies indicated that increased tea consumption did not yield any significant impact on renal function improvement or delay in nephropathy progression^14^. However, a cohort study reported that oolong tea could promote the efficiency of eGFR compared to green tea and black tea^19^. In addition, it is reported that bioactive substances that existed in tea may improve risk factors for renal insufficiency, including blood pressure, oxidative stress, dyslipidemia, and insulin resistance or hyperglycemia^21^. In summary, whether tea consumption was inversely associated with CKD risk remains unclear.

The existing body of research has reported inconsistent results, making it difficult to draw definitive conclusions. Few studies have examined the relationship between different sources of caffeine intake and CKD risk. Most research has focused on coffee consumption’s impact on renal function, with only a handful examining the link between caffeine metabolites and renal function^22^. Moreover, earlier investigations into the long-term effects of caffeine have been hindered by the considerable error inherent in self-reported caffeine intake^23^. Urine caffeine metabolites are reliable markers for evaluating caffeine intake from food sources and can effectively avoid the above - mentioned limitations^24^.

To partially address these gaps, the present study adopts a cross-sectional design, utilizing data from the National Health and Nutrition Examination Survey (NHANES) 1999–2018. This allows us to assess the cross-sectional associations between coffee, tea, caffeine consumption and urine caffeine metabolites and CKD among adults in the United States.

## Materials and methods

### Data collection and study population

The data utilized in this study are obtained from the National Health and Nutrition Examination Survey (NHANES). NHANES was conducted by the Centers for Disease Control and Prevention of America, which is a two-year-cycle cross-sectional survey and aims at evaluating the health and nutritional status of American population^25^. Initially, participants were interviewed at their home to gather essential background information, such as socio-demographic details, medical history, and family history. Later on, they attended a mobile examination center (MEC) to provide additional data such as anthropometric measurements, blood pressure, laboratory tests, and other relevant information.

**Fig. 1:**
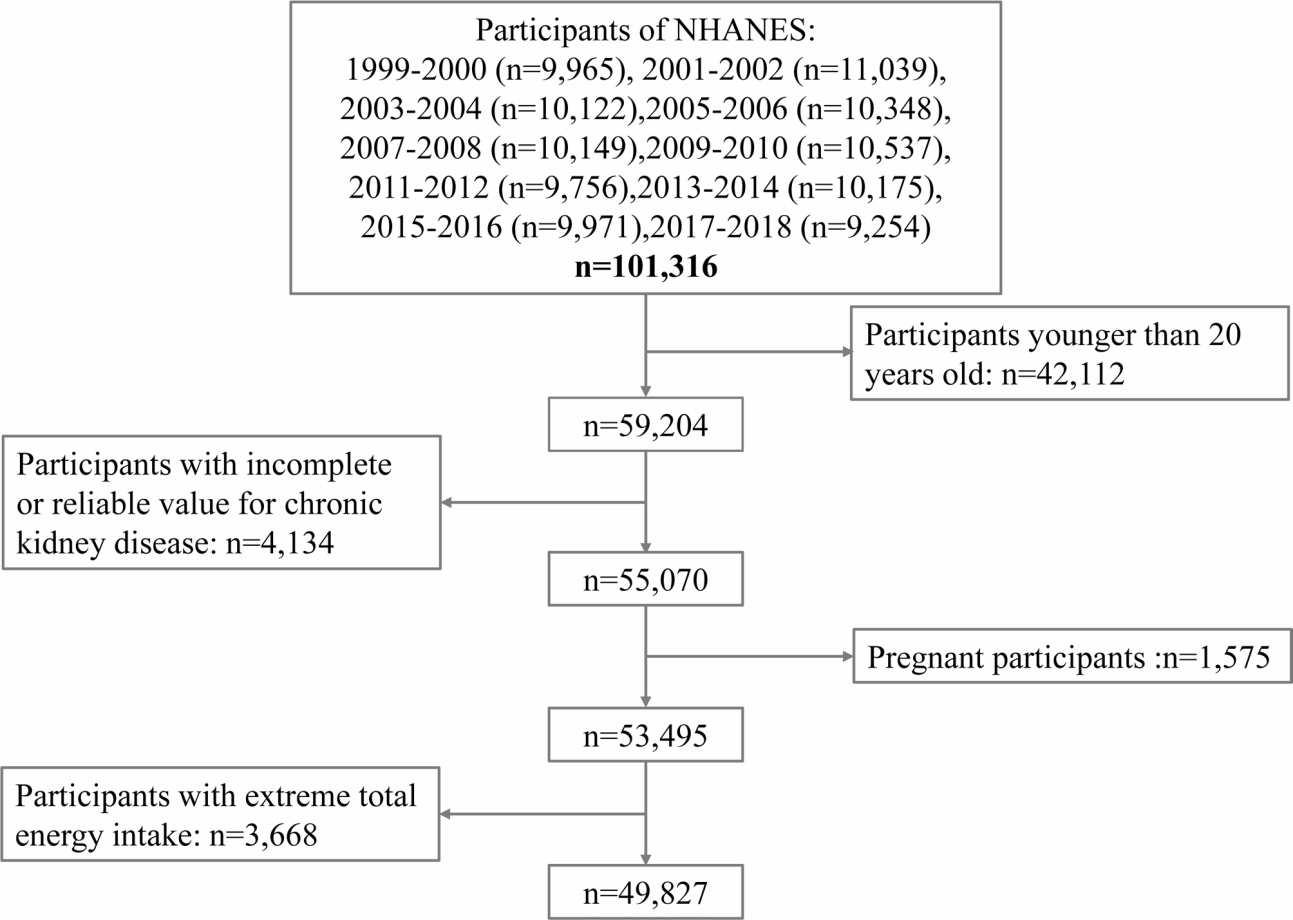
Flowchart of the screening process for the selection of eligible participants.

Ten cycles (1999–2000, 2001–2002, 2003–2004, 2005–2006, 2007–2008, 2009–2010, 2011–2012, 2013–2014, 2015–2016, 2017–2018) data included this study were downloaded from the NHANES website. A total of 101,316 participants from 1999 to 2018 enrolled in the survey. We excluded participants based on the following criteria: aged under 20 years old (*n* = 42,112), incomplete or unreliable CKD data (*n* = 4,134), pregnant (*n* = 1,575), and extreme total energy intakes (< 500 or > 8000 kcal/day for men, < 500 or > 5000 kcal/day for women) (*n* = 3,668)^26^. After exclusions,49,827 individuals were included in the final analysis (Fig. 1). NHANES was approved by the National Center for Health Statistics Research Ethics Review Board.

### Chronic kidney disease assessment

CKD was defined as eGFR < 60 ml/min/1.73m^2^ or persistent albuminuria^27^. The eGFR was calculated using the 2021 chronic kidney disease-Epidemiology Collaboration Eq. (2021 CKD-EPI): eGFR = 142 × min(SCr/κ,1)^α × max(SCr/κ,1)^−1.209 × 0.9938^Age × 1.012 [if female], where SCr is serum creatinine in mg/dL, κ is 0.7 for females and 0.9 for males, α is −0.241 for females and − 0.303 for males, min denotes the minimum of SCr/κ or 1, max indicates the maximum of SCr/κ or 1, and Age is in years^27,28^. Albuminuria was defined as albumin-to-creatinine ratio ≥ 30 mg/g^27,28^. We did not define persistent proteinuria for most participants only measured once in the survey, and for the timing of second urine collection varied (first morning void vs. random) when albuminuria was repeated during selected cycles of NHANES^29^.

### Exposure assessment

Frequency of coffee consumption, tea consumption, total caffeine consumption, caffeine from coffee, and caffeine from tea was obtained from two 24-hour dietary recall interviews, which were conducted by trained interviewers using an automated data collection system during the MEC examination^30^. Consumption of total caffeine and caffeine from tea and coffee was segmented into tertiles. Consumption of coffee and caffeine from coffee was divided into three categories. Participants with no consumption of coffee and caffeine from coffee were classified into group 1 (intake = 0), and individuals with consumption were divided into group 2 (< median) and group 3 (≥ median) based on its median intake (g/d) among the participants with consumption. Intake of tea and caffeine from tea was categorized into two groups. No consumption of tea and caffeine from tea was classified into group 1 (intake = 0), individuals with consumption were divided into group 2.

The measurement of urinary caffeine and its metabolite levels has been suggested as a reliable method for evaluating caffeine consumption^31^. Thus, we also evaluated the association of urinary caffeine metabolites and CKD using three cycles (2009–2010, 2011–2012, 2013–2014) data. All NHANES participants were required to provide urine samples in a mobile examination center (MEC). Ultra-high performance liquid chromatography-electrospray ionization-tandem quadrupole mass spectrometry was employed for the analysis of caffeine and its metabolites^32^including 1-methyluric acid (1U), 3-methyluric acid (3U), 7-methyluric acid (7U), 1,3-dimethyluric acid (13U), 1,7-dimethyluric acid (17U), 3,7-dimethyluric acid (37U), 1,3,7-trimethyluric acid (137U), 1-methylxanthine (1X), 3-methylxanthine (3X), 7-methylxanthine(7X), theophylline(13X), paraxanthine(17X), theobromine(37X), caffeine(137X) and5-acetylamino-6-amino-3-methy luracil (AAMU). Spearman correlation analysis revealed that all 15 metabolites exhibited a positive correlation with caffeine intake. Notably, 9 of these metabolites demonstrated a moderate positive correlation with caffeine intake (Spearman r, 0.501–0.578, *P* < 0.001) (**Table ****S1**). Therefore, we only assessed the relationship between 9 urinary caffeine metabolites and CKD. Bonferroni correction was applied to account for multiple comparisons, with a significance level of *P* < 0.05/9 for the nine metabolites assessed.

### Covariates

Information of socio-demography, lifestyle, and health related factors had been collected by an interviewer using the Sample Person and Family Demographics questionnaires. Data included sex (male and female), age (20–39 years, 40–59 years, and ≥ 60 years), race (Mexican American, Other Hispanic, Non-Hispanic White, Non-Hispanic Black, and other races), educational level (below high school, High school, and above high school), marital status (married/living with partner, and widowed/divorced/separated/never married), body mass index (BMI) (normal: <25 kg/m^2^, overweight: 25 to < 30 kg/m^2^, obese: ≥30 kg/m^2^), physical activity (vigorous, moderate, and other), family income (Under $20,000, and $20,000 and over), smoking behavior (smoked at least 100 cigarettes in life or not), hypertension prevalence rate, diabetes mellitus prevalence rate, alcohol use (had at least 12 alcohol drinks/year or not), and total energy intake. We used the “dietaryindex” R package to calculate the AHEI based on dietary information^33^.

### Statistical analysis

Statistical analysis was conducted using Stata 15.0 (Stata Corporation, College Station, TX). A new sample weight (the original 2-year sample weight divided by 2) had been constructed according to the analytical guidelines of NHANES when combing ten 2-year cycles of the continuous data^34^. In brief, we utilize the 4-year sample weights provided by NCHS instead of the 2-year sample weights. We conducted the Kolmogorov–Smirnov normality test to test the normality of continuous variables, and we described continuous variables with mean ± standard deviation (SD) (normally distributed) or median (interquartile range) (non-normally distributed). Student’s t-test (normally distributed) and the Mann–Whitney U test was used to examine the differences of continuous variable between the CKD group and the non-CKD group. Chi-square tests were chosen to examine the difference of categorical variables between the different groups.

In this study, we conducted binary logistic regression analyses to evaluate the association of coffee consumption, tea consumption, caffeine consumption and urinary caffeine and caffeine metabolite with CKD. Two models had been adopted to evaluate the association of coffee consumption, tea consumption, and caffeine consumption with CKD. Model 1, adjusted for age and sex; model 2 (fully adjusted model), adjusted for age, sex, race, marital status, educational level, family income, body mass index, physical activity, smoking status, alcohol consumption, hypertension, diabetes, alternative healthy eating index and energy intake. In addition, we performed subgroup analyses stratified by sex (male and female) and age (< 60 and 60~) to test sex and age as an interaction with coffee consumption, tea consumption, and caffeine consumption in the model that adjusted for the same covariates. As hypertension and diabetes may lie on the causal pathway between coffee/tea consumption and CKD, we conducted a sensitivity analysis excluding them. In addition, liner regression was conducted to evaluate the association of coffee consumption, tea consumption, and caffeine consumption with eGFR. A two-sided *p* < 0.05 was considered statistically significant.

## Results

### General characteristics

The baseline characteristics are presented in Table 1. A total of 49,827 individuals (mean age: 47.9 ± 19.2 years old) was included in this study, among whom 8,554 (17.2%) were diagnosed with CKD. Participants with CKD demonstrated a higher likelihood of being older, female, non-Hispanic Black individuals, having a lower educational attainment, being widowed/divorced/separated/never married, overweight, physically inactive, possessing lower family income and exhibiting a higher prevalence of hypertension and diabetes compared to those without CKD. Additionally, individuals with CKD reported higher coffee consumption but lower caffeine and energy intake.

**Table 1:** Characteristics of the study population, National health and nutrition examination survey (NHANES) 1999–2018.

	Number of subjects (*N*)	With CKD	Without CKD	*P* Value
Number of subjects (%)	49,827 (100.0)	8,554 (17.2)	41,273(82.8)	
Age, mean (standard deviation)^b^	47.9 (19.2)	61.92 (18.2)	44.99 (18.0)	< 0.001
Sex (%) ^a^				< 0.001
Male	24,900 (50.0)	4,046 (47.3)	20,854 (50.5)	
Female	24,927 (50.0)	4,508 (52.7)	20,419 (49.5)	
Race (%) ^a^				< 0.001
Mexican American	9,093 (18.2)	1,305 (15.3)	7,788 (18.9)	
Other Hispanic	4,047 (8.1)	583 (6.8)	3,464 (8.4)	
Non-Hispanic White	21,775 (43.7)	3,915 (45.8)	17,860 (43.3)	
Non-Hispanic Black	10,639 (21.4)	2,197 (25.7)	8,442 (20.5)	
Other races	4,273 (8.6)	554 (6.5)	3,719 (9.0)	
Educational level (%) ^a^				< 0.001
Below high school	13,753 (27.6)	2,979 (34.8)	10,774 (26.1)	
High school	11,988 (24.1)	2,082 (24.3)	9,906 (24.0)	
Above high school	24,086 (48.3)	3,493 (40.8)	20,593 (49.9)	
Material status (%) ^a^				< 0.001
Married/living with partner	27,730 (55.7)	3,955 (46.2)	16,096 (39.0)	
Widowed/divorced/separated/never married	20,051 (40.2)	4,412 (51.6)	23,318 (56.5)	
Unknown	2,046 (4.1)	187 (2.2)	1,859 (4.5)	
Body mass index (%) ^a^				< 0.001
< 25 kg/m ^2^	15,460 (31.0)	2,213 (25.9)	13,247 (32.1)	
25–30 kg/m ^2^	16,273 (32.7)	2,590 (30.3)	13,683 (33.2)	
≥ 30 kg/m ^2^	18,094 (36.3)	3,751 (43.9)	14,343 (34.8)	
Physical activity (%) ^a^				< 0.001
Vigorous	17,549 (35.2)	1,703 (19.9)	15,846 (38.4)	
Moderate	15,073 (30.3)	2,819 (33.0)	12,254 (29.7)	
Other	17,205 (34.5)	4,032 (47.1)	13,173 (31.9)	
Family income (%) ^a^				< 0.001
Under $20,000	13,101 (26.3)	2,871 (33.6)	10,230 (24.8)	
$20,000 and over	31,532 (63.3)	4,983 (58.3)	26,549 (64.3)	
Unknown	5,194 (10.4)	700 (8.2)	4,494 (10.9)	
Smoked at least 100 cigarettes in life (%) ^a^	21,486 (43.1)	4,186 (48.9)	17,300 (41.9)	< 0.001
-Hypertension (%) ^a^	25,880 (51.9)	6,689 (78.2)	19,191 (46.5)	< 0.001
Diabetes(%) ^a^	5,717 (11.5)	2,504 (29.3)	3,213 (7.8)	< 0.001
Had at least 12 alcohol drinks/year (%) ^a^	30,467 (61.1)	4,933 (57.7)	25,534 (61.9)	< 0.001
Total energy intake (kcal/d), median (interquartile range)^b^	2,067.24 (1,064.0)	1,818.79 (764.7)	2,118.73 (885.3)	< 0.001
Tea intake (g/day), median (interquartile range)^b^	151.41 (177.6)	140.92 (325.4)	153.59 (367.4)	0.137
Coffee intake (g/day), median (interquartile range)^b^	253.35 (370.0)	264.80 (370.5)	250.98 (407.5)	< 0.001
Caffeine (mg/day), median (interquartile range)^b^	142.83 (173.5)	104.27 (155.8)	111.14 (174.4)	< 0.001

Data is number of subjects (percentage), medians (interquartile ranges) or mean (standard deviation).

^a^ Chi-square test was used to compare the percentage between participants with and without CKD.

^**b**^ Mann-Whitney U test was used to compare the median values between participants with and without CKD.

### The association of coffee consumption with CKD

**Fig. 2:**
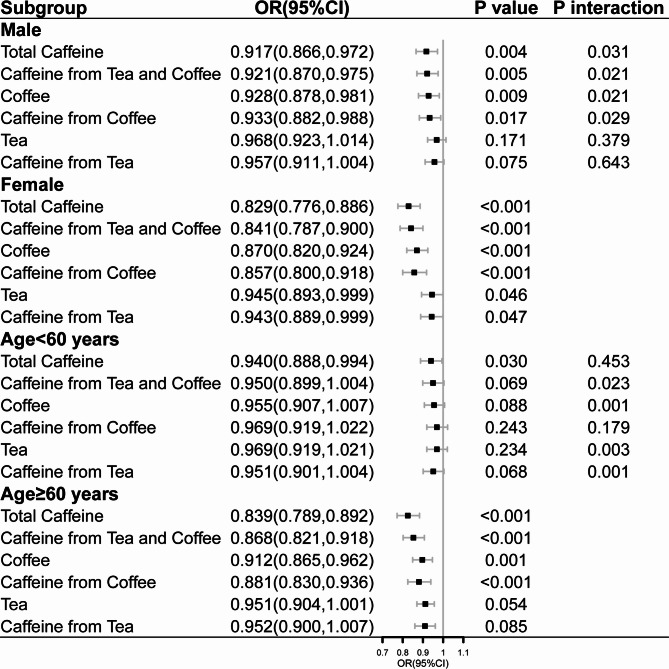
Association of coffee, tea, and caffeine consumption with chronic kidney disease. The ORs were adjusted for potential confounders, including age (in sex group), sex (in age group), race, marital status, educational level, family income, body mass index, physical activity, smoking status, alcohol consumption, hypertension, diabetes, alternative healthy eating index and energy intake. The interaction terms were evaluated through likelihood tests. CI, confidence interval; OR, odds ratio. P interaction indicates the test for interaction terms by subgroup variables (sex and age).CI, confidence interval; OR, odd ratio. P interaction indicates the test for interaction terms by subgroup variables (sex and age).

There was a significant inverse association between coffee consumption and CKD **(**Table 2). In model 2, compared to individuals who reported no coffee consumption, those who reported consuming 0.01–352.5 g/day exhibited an odds ratio (OR) of 0.918 (95% confidence interval [CI]: 0.845–0.998; *P* = 0.044), while those who consumed more than 352.5 g/day had an OR of 0.760 (95% CI: 0.701–0.823; *P* < 0.001). Similarly, the OR for CKD was 0.902(95%CI :0.868–0.938; *P* < 0.001) in response to per SD increment in coffee consumption. The findings of the analyses, which were stratified by sex and age, exhibited consistent patterns (Fig. 2). A statistically significant association between coffee consumption and CKD was observed in females (*P* interaction = 0.021). Furthermore, a stronger association between coffee consumption and CKD was found among participants aged over 60 years (*P* interaction = 0.001). The results remained stable after sensitivity analysis.

**Table 2:** Weighted odds ratios (95% confidence intervals) for CKD across tea, coffee and caffeine intakes.

	Cases/Control	Model 1^a^		Model 2^b^	
		OR (95% CIs)	*P* value	OR (95% CIs)	*P* value
**Coffee (g/day)** ^**c**^					
0	3,298/17,902	1 (Ref)		1 (Ref)	
0.01 ~ 352.5	2,766/10,442	0.885(0.815,0.961)	0.004	0.918(0.845,0.998)	0.044
≥ 352.5	2,490/10,894	0.652(0.602,0.706)	< 0.001	0.760(0.701,0.823)	< 0.001
1 SD increment		0.853(0.819,0.888)	< 0.001	0.902(0.868,0.938)	< 0.001
**Tea (g/day)** ^**c**^					
0	5,813/26,833	1 (Ref)		1 (Ref)	
> 0	2,741/12,405	0.860(0.797,0.929)	< 0.001	0.913(0.843,0.989)	0.025
1 SD increment		0.937(0.898,0.977)	0.003	0.955(0.919,0.993)	0.020
**Total Caffeine (mg/day)** ^**c**^					
< 44.5	3,007/12,699	1 (Ref)		1 (Ref)	
44.5 ~ 153.00	2,990/12,963	0.884(0.820,0.952)	0.001	0.933(0.862,1.010)	0.054
≥ 153.00	2,557/13,576	0.640(0.591,0.692)	< 0.001	0.734(0.674,0.799)	< 0.001
1 SD increment		0.835(0.800,0.873)	< 0.001	0.876(0.840,0.914)	< 0.001
**Caffeine from Tea and Coffee (mg/day)** ^**c**^					
< 2.01	2,513/13,298	1 (Ref)		1 (Ref)	
2.01 ~ 111.59	3,245/12,633	0.842(0.768,0.923)	< 0.001	0.903(0.822,0.993)	0.036
≥ 111.59	2,796/13,307	0.625(0.577,0.677)	< 0.001	0.748(0.686,0.814)	< 0.001
1 SD increment		0.836(0.800,0.873)	< 0.001	0.886(0.851,0.923)	< 0.001
**Caffeine from Coffee (mg/day)** ^**c**^					
0	3,302/17,928	1 (Ref)		1 (Ref)	
0.01 ~ 109.50	2,911/10,242	0.878(0.808,0.956)	0.003	0.913(0.839,0.994)	0.036
≥ 109.50	2,341/11,068	0.657(0.607,0.710)	< 0.001	0.766(0.707,0.829)	< 0.001
1 SD increment		0.851(0.815,0.889)	< 0.001	0.901(0.865,0.938)	0.001
**Caffeine from Tea (mg/day)** ^**c**^					
0	6,016/27,898	1 (Ref)		1 (Ref)	
> 0	2,538/11,340	0.875(0.811,0.945)	0.001	0.925(0.854,1.001)	0.053
1 SD increment		0.934(0.895,0.975)	0.002	0.948(0.911,0.987)	0.010

Calculated using binary logistic regression.

^a^ Model 1 adjusted for age and sex.

^b^ Model 2 additionally adjusted for race, marital status, educational level, family income, body mass index, physical activity, smoking status, alcohol consumption, hypertension, diabetes, alternative healthy eating index and total daily energy intake (continuous, kcal/d).

^c^ Intake of total caffeine and caffeine from tea and coffee was segmented into tertiles. Intake of coffee and caffeine from coffee was divided into three categories. Participants with no consumption of coffee and caffeine from coffee were classified into group 1 (intake = 0), and individuals with consumption were divided into group 2 (< median) and group 3 (≥ median) based on its median intake (g/d) among the participants with consumption. Intake of tea and caffeine from tea was categoried into two group. No consumption of tea and caffeine from tea was classified into group 1 (intake = 0), individuals with consumption were divided into group 2.

### The association of tea consumption with CKD

We observed a significant inverse association between tea consumption and CKD (Table 2). Compared to those who reported no tea consumption, individuals who consumed tea had a multivariate-adjusted OR of 0.913 (95% CI: 0.843–0.989; *P* = 0.025) for CKD. In addition, the OR for CKD was 0.955(95%CI:0.919–0.993; *P* = 0.020) in response to per SD increment in tea consumption. Stratification analyses based on sex and age consistently demonstrated similar patterns as the primary analysis (Fig. 2). Notably, no significant interaction effect of sex or age on the association between tea consumption and CKD was observed (*P* interaction > 0.05). The results remained stable after sensitivity analysis (**Table S2**).

### The association of caffeine consumption with CKD

Caffeine consumption showed a significant inverse association with CKD (Table 2). Compared to individuals reporting a consumption of less than 44.5 g/day, those who reported consuming 44.5–153.0 g/day had an OR of 0.933 (95%CI: 0.862–1.010; *P* = 0.054), while those consuming more than 153.0 g/day had an OR of 0.734 (95% CI: 0.634–0.799; *P* < 0.001). Similarly, the OR for CKD was 0.876(95%CI: 0.840–0.914; *P* < 0.001) in response to per SD increment in caffeine consumption. The inverse association remained significant in the subgroup analyses stratified by sex and age (Fig. 2). Sex acted as a modifier to the effect of caffeine on CKD (*P* interaction = 0.031), while age did not modify the association (*P* interaction = 0.453). The results remained stable after sensitivity analysis (**Table S2**).

We found that coffee-sourced caffeine was significantly associated with CKD (OR for 1 SD increment:0.901, 95%CI: 0.865–0.938, *P* = 0.001) (Table 2). Sex serves as effect modifiers in the relationship between caffeine from coffee consumption and CKD (*P* interaction = 0.029) (Fig. 2). Tea-sourced caffeine was slightly associated with CKD (OR for 1 SD increment:0.948, 95%CI:0.911–0.987, *P* = 0.010) (Table 2). The association between tea-sourced caffeine and CKD was modified by age (*P* interaction = 0.001) (Fig. 2). In addition, an inverse association was observed between caffeine intake from tea and coffee and the odds of CKD (OR for 1 SD increment:0.886, 95%CI:0.851–0.923, *P* < 0.001) (Table 2). In the stratified analyses, a significantly stronger association was observed among females and older individuals (*P* interaction < 0.05) (Fig. 2). The results remained stable after sensitivity analysis (Table S2).

### Associations of tea, coffee and caffeine intakes with eGFR

The associations between tea, coffee, and caffeine intakes with eGFR are presented in Table 3. A statistically significant increase of 0.229 in eGFR per 100 mg/day increment in total caffeine consumption was observed (*β* = 0.229, *P* < 0.001). Similarly, a statistically significant increase of 0.242 per 100 mg/day increment in eGFR was found for caffeine derived from coffee and tea consumption (*β* = 0.242, *P* < 0.001). Furthermore, an increase of 0.273 per 100 mg/day increment in eGFR was noted for caffeine sourced specifically from coffee (*β* = 0.273, *P* < 0.001). Additionally, each additional intake of coffee by the amount of 100 g/day was associated with a modest increase of eGFR by approximately 0.067 units. However, no significant association between tea or tea-sourced caffeine and eGFR was observed (all *P* > 0.05).

**Table 3:** Associations of tea, coffee and caffeine intakes with estimated glomerular filtration rate.

	Model 1^a^			Model 2 ^b^		
	Beta ^*^	SD	*P*	Beta ^*^	SD	*P*
Total Caffeine	0.309	0.054	< 0.001	0.229	0.050	< 0.001
Caffeine from Tea and Coffee	0.329	0.054	< 0.001	0.242	0.050	< 0.001
Coffee	0.123	0.023	< 0.001	0.067	0.022	0.003
Caffeine from Coffee	0.387	0.056	< 0.001	0.273	0.053	< 0.001
Tea	−0.030	0.025	0.232	−0.014	0.023	0.542
Caffeine from Tea	−0.135	0.148	0.363	−0.024	0.137	0.859

^*^ The beta coefficient was calculated for each 100 mg/day increase in caffeine consumption, including total caffeine intake, caffeine from tea and coffee, caffeine from coffee alone, and caffeine from tea. Additionally, 100 g/day increment for coffee and tea intake. The beta value represents the change in eGFR (ml/min/1.73m2) per 100 g/day increase in coffee and tea intake and per 100 mg/day increase in caffeine consumption.

^a^ Model 1 adjusted for age and sex.

^b^ Model 2 additionally adjusted for race, marital status, educational level, family income, body mass index, physical activity, smoking status, alcohol consumption, hypertension, diabetes, alternative healthy eating index and total daily energy intake (continuous, kcal/d).

### Association of urinary caffeine metabolites and CKD

We also evaluated the association between urinary caffeine metabolites and CKD (**Table S3**). We observed an inverse relationship between 17X and CKD risk (OR: 0.994, 95%CI: 0.990–0.999, *P* = 0.023), when accounting for multiple comparisons, the association no longer remains statistically significant. No significant associations were found between other urinary caffeine metabolites and CKD.

## Discussion

In the present large cross-sectional study of US adults, there was a significant inverse association between consumption of coffee, tea and caffeine and CKD. These associations were robustly observed even after adjusted for potential mediators such as hypertension, and diabetes. Additionally, our findings suggest a positive correlation between the consumption of total caffeine, coffee-derived caffeine, and coffee with eGFR levels.

The protective effect of coffee on renal function has been a topic of extensive research. Our findings align with previous studies that have reported a beneficial role of coffee in renal health^12,35^. However, the literature is not entirely consistent, with some studies showing no significant association between coffee consumption and CKD risk^14,18^. This discrepancy may be attributed to differences in study design, population characteristics, and the control of confounding variables. A meta-analysis included four observational studies reported no impact of coffee consumption on CKD risk in males but found a decreased risk in females^36^. Another meta-analysis of cohort study reported that consumption of coffee was significantly associated with a lower risk of CKD^37^. In addition, a Mendelian Randomization (MR) study investigated the impact of coffee on the renal function and reported a beneficial effect of coffee consumption on renal function^38^. These findings collectively suggest that coffee may have a protective effect, particularly in certain subpopulations.

The beneficial effect of coffee may be attributed to the anti-oxidant and anti-inflammatory properties of coffee^39,40^. Oxidant stress (OS) is characterized by the pro-/antioxidant balance, could influence the function of cells and tissues due to the excessive generation of highly reactive oxygen (ROS)^41^. The kidney is an important metabolic organ, rich in oxidation reactions in mitochondria, which makes it vulnerable to damage caused by OS, and OS can accelerate the development of kidney disease^41^. Coffee contains various compounds, including hydroxyhydroquinone, and caffeine, which have known anti-oxidant effects^42^. In addition, increasing evidence showed that coffee exerted the protective effect by against OS^39^. A randomized controlled trail study showed that coffee consumption might increase the antioxidant capacity of plasma^43^. Given the role of OS in CKD development and the anti-inflammatory properties of coffee, it is plausible that coffee may contribute to CKD prevention.

The association between tea consumption and renal function has been extensively studied, while the results are inconsistent. A cohort study reported that there was no significant association between tea consumption and the change of eGFR^18^while another cohort study reported that high tea consumption was related to a decline of eGFR in 1 year^44^. In our study, we found that tea consumption might decrease the risk of CKD, whereas a study reported that there was no significant association between high tea consumption and CKD risk^14^. In addition, a MR study demonstrated that causal relationships existed between increased tea consumption and reduced risk of CKD and albuminuria and increased eGFR^45^. The protective effect of tea is likely due to its rich content of bioactive substances, such as phenolic compounds and minerals with antioxidant and anti-inflammatory properties^46^. Tea catechins, including epallocateigchin-3-gallate (EGCG), epigallocatechin, and epicatechin, may improve endothelial function and arterial vasodilation by reducing ROS levels^47,48^. These catechins act as metal ion chelators and ROS scavengers, and they induce antioxidant enzymes while inhibiting pro-oxidant enzymes^47,48^. Several animals and in vitro studies suggested that EGCG had potential effects on the renal function. Animal studies have shown that EGCG can ameliorate renal damage through antioxidant, anti-inflammatory, and apoptosis-inducing effects^49,50^.

Our study also observed an inverse association between caffeine consumption and CKD risk, as well as a positive association with eGFR levels. These results are supported by previous research showing that genetically predicted caffeine intake is associated with increased eGFR and reduced CKD risk^16,51^. The modulation of changes in eGFR by caffeine has been suggested to occur through diuresis and natriuresis via binding adenosine receptors, interference with the anti-inflammatory effects of adenosine, as well as stimulation of key proliferative mechanisms involved in glomerular remodeling and sclerosis^52^. This suggests that caffeine’s effects on renal function may be multifaceted, involving both hemodynamic and metabolic pathways.

The main advantage of this study is the data source. The data from NHANES with continuous quality assurance and quality control processes, therefore the data are timely and of high quality. They have employed population-based cluster random selection to identify a sample that is nationally representative, thus the data from NHANES can be extrapolated to the entire US population. Additionally, we controlled wide ranges of potential confounders to provide a better estimation of the association of coffee, tea, and caffeine intake with CKD risk.

Our study has several limitations. First, the cross-sectional design precludes establishing causality and exploring the temporal dynamics of the associations between tea, coffee, or caffeine consumption and CKD risk. To address this, we reviewed existing longitudinal studies and meta-analyses that support the robustness of our findings across different study designs. Second, dietary data were obtained from two 24-hour recall interviews, which may not accurately reflect long-term dietary intake. Future studies should consider using multiple 24-hour recalls or food diaries to better capture long-term dietary patterns. Additionally, dietary recording apps could enhance data accuracy and continuity. Finally, we did not account for variations in caffeine content due to differences in coffee bean type, origin, and processing methods. Future studies should consider these factors to provide a more comprehensive understanding of the relationship between coffee consumption and CKD risk.

### Clinical implications

Our findings have several clinical implications. Given the inverse association between coffee, tea, and caffeine consumption and CKD risk, healthcare providers may consider discussing these dietary factors with patients at risk for kidney disease. Encouraging moderate consumption of coffee or tea could potentially serve as a simple, low-cost adjunct to traditional CKD prevention strategies. However, it is important to consider individual patient factors and not to replace established treatments. Further research is needed to determine optimal consumption levels and to confirm associations these in diverse populations.

## Conclusions

In conclusion, we found that tea, coffee or caffeine consumption was inversely associated with CKD, which may be attributed to the anti-oxidant and anti-inflammatory properties of the bioactive substances of coffee and tea. Our study may unravel the effect of coffee and tea consumption on CKD development. Large-scale, prospective studies are warranted to further validate the protective effects of tea, coffee or caffeine consumption on CKD risk in adults.

## Electronic supplementary material

Below is the link to the electronic supplementary material.

Supplementary Material 1

## Data Availability

The original data were retrieved from https://www.cdc.gov/nchs/nhanes/index.htm. All data generated or analysed during this study are included in this published article.
