# Supplementary material for: Association between coffee and caffeine consumption and chronic kidney disease
Source: Sci Rep. 2025 Jul 12;15:25228. doi: 10.1038/s41598-025-11543-4 (PMC12255770; doi:10.1038/s41598-025-11543-4)
Supplement: Supplementary file 1 — Supplementary Material 1 [file 41598_2025_11543_MOESM1_ESM.docx]

Association of coffee, tea, and caffeine consumption with chronic kidney disease: analysis of NHANES 1999-2018

Panpan Gao ^1^, Xinrong Ji ^2^, Weiwei Wang ^2^, Yao Chen ^2^, Zhan Gao ^2^, Zengli Yu ^1, 4*^

**Supplementary Tables**

**Table S1** Spearman Correlation Coefficients Between Urinary Metabolites and Caffeine Intake.

**Table S2** Weighted odds ratios (95% confidence intervals) for CKD across tea, coffee and caffeine intakes excluded subjects with diabetes and hypertension.

**Table S3** Weighted odds ratios (95% confidence intervals) for CKD across urinary caffeine metabolites.

Table S1 Spearman Correlation Coefficients Between Urinary Metabolites and Caffeine Intake

| Metabolites | Spearman r | P |
| --- | --- | --- |
| 1-methyluric acid | 0.560 | <0.001 |
| 3-methyluric acid | 0.333 | <0.001 |
| 7-methyluric acid | 0.320 | <0.001 |
| 1,3-dimethyluric acid | 0.578 | <0.001 |
| 1,7-dimethyluric acid | 0.549 | <0.001 |
| 3,7-dimethyluric acid | 0.267 | <0.001 |
| 1,3,7-trimethyluric acid | 0.501 | <0.001 |
| 1-methylxanthine | 0.569 | <0.001 |
| 3-methylxanthine | 0.316 | <0.001 |
| 7-methylxanthine | 0.311 | <0.001 |
| 1,3-dimethylxanthine (theophylline) | 0.550 | <0.001 |
| 1,7-dimethylxanthine (paraxanthine) | 0.552 | <0.001 |
| 3,7-dimethylxanthine (theobromine) | 0.262 | <0.001 |
| 1,3,7-trimethylxanthine (caffeine) | 0.529 | <0.001 |
| AAMU | 0.570 | <0.001 |

**Table S2 Weighted odds ratios (95% confidence intervals) for CKD across tea, coffee and caffeine intakes excluded subjects with diabetes and hypertension.**

|  | Cases/ Control | Model 1^a^ |  | Model 2^b^ |  |
| --- | --- | --- | --- | --- | --- |
|  |  | OR (95% CIs) | *P* value | OR (95% CIs) | *P* value |
| **Coffee (g/day)^c^** |  |  |  |  |  |
| 0 | 2,979/ 19,362 | 1 (Ref) |  | 1 (Ref) |  |
| 0.01~352.5 | 2,447/ 11,288 | 0.892(0.755,1.053) | 0.176 | 0.926(0.783,1.094) | 0.044 |
| ≥352.5 | 2,170/ 11,581 | 0.681(0.571,0.814) | <0.001 | 0.743(0.613,0.901) | <0.001 |
| 1 SD increment |  | 0.854(0.784,0.929) | <0.001 | 0.878(0.806,0.956) | 0.003 |
| **Tea (g/day)^c^** |  |  |  |  |  |
| 0 | 5,213/ 28,848 | 1 (Ref) |  | 1 (Ref) |  |
| >0 | 2,383/ 13,383 | 0.952(0.828,1.095) | 0.490 | 0.987(0.857,1.136) | 0.025 |
| 1 SD increment |  | 0.909(0.849,0.972) | 0.006 | 0.922(0.861,0.987) | 0.020 |
| **Total Caffeine (mg/day)^c^** |  |  |  |  |  |
| <44.5 | 2,694/ 13,874 | 1 (Ref) |  | 1 (Ref) |  |
| 44.5~153.00 | 2,657/ 13,983 | 0.819(0.708,0.947) | 0.007 | 0.85(0.732,0.986) | 0.054 |
| ≥153.00 | 2,245/ 14,374 | 0.626(0.535,0.733) | <0.001 | 0.673(0.565,0.801) | <0.001 |
| 1 SD increment |  | 0.818(0.747,0.895) | <0.001 | 0.837(0.763,0.918) | <0.001 |
| **Caffeine from Tea and Coffee (mg/day)^c^** |  |  |  |  |  |
| <2.01 | 2,292/ 12,372 | 1 (Ref) |  | 1 (Ref) |  |
| 2.01~111.59 | 2,866/ 13,720 | 0.851(0.726,0.999) | 0.048 | 0.881(0.751,1.033) | 0.036 |
| ≥111.59 | 2,438/ 14,139 | 0.657(0.556,0.777) | <0.001 | 0.727(0.607,0.871) | <0.001 |
| 1 SD increment |  | 0.836(0.764,0.913) | <0.001 | 0.862(0.79,0.942) | 0.001 |
| **Caffeine from Coffee (mg/day)^c^** |  |  |  |  |  |
| 0 | 2,982/ 19,391 | 1 (Ref) |  | 1 (Ref) |  |
| 0.01~109.50 | 2,567/ 11,105 | 0.857(0.724,1.014) | 0.072 | 0.88(0.742,1.044) | 0.036 |
| ≥109.50 | 2,047/ 11,735 | 0.719(0.601,0.861) | <0.001 | 0.793(0.654,0.961) | <0.001 |
| 1 SD increment |  | 0.87(0.797,0.95) | 0.002 | 0.896(0.823,0.976) | 0.012 |
| **Caffeine from Tea (mg/day)^c^** |  |  |  |  |  |
| 0 | 5,391/ 29,993 | 1 (Ref) |  | 1 (Ref) |  |
| >0 | 2,205/ 12,238 | 0.931(0.811,1.07) | 0.314 | 0.959(0.831,1.107) | 0.053 |
| 1 SD increment |  | 0.881(0.818,0.95) | 0.001 | 0.894(0.828,0.965) | 0.004 |

**Table S3 Weighted odds ratios (95% confidence intervals) for CKD across urinary caffeine metabolites.**

| Variables | Model 1^a^ | | Model 2 ^b^ | |
| --- | --- | --- | --- | --- |
|  | OR (95% CI) | P | OR (95% CIs) | P |
| 1-methyluric acid, umol/L | 1.000(1.000,1.001) | 0.433 | 1.000(1.000,1.001) | 0.481 |
| 1,3-dimethyluric acid, umol/L | 1.000(0.999,1.001) | 0.759 | 1.000(0.999,1.001) | 0.504 |
| 1,7-dimethyluric acid, umol/L | 1.000(0.999,1.002) | 0.488 | 1.000(0.999,1.002) | 0.591 |
| 1,3,7-trimethyluric acid, umol/L | 1.002(0.982,1.022) | 0.858 | 0.999(0.976,1.023) | 0.922 |
| 1-methylxanthine, umol/L | 0.998(0.997,1.000) | 0.025 | 0.998(0.997,1.000) | 0.072 |
| 1,3-dimethylxanthine (theophylline), umol/L | 0.998(0.992,1.004) | 0.552 | 1.000(0.994,1.005) | 0.856 |
| 1,7-dimethylxanthine (paraxanthine), umol/L | 0.993(0.989,0.997) | 0.001 | 0.994(0.990,0.999) | 0.023 |
| 1,3,7-trimethylxanthine (caffeine), umol/L | 0.999(0.99,1.008) | 0.855 | 1.001(0.990,1.011) | 0.907 |
| AAMU, umol/L | 1.000(1.000,1.001) | 0.392 | 1.000(1.000,1.001) | 0.703 |

Bonferroni correction was employed to address the issue of multiple testing, given the number of metabolites. P< 0.05/9 was deemed significant.

^a^ Model 1 adjusted for age and sex.

^b^ Model 2 additionally adjusted for race, marital status, educational level, family income, body mass index, physical activity, smoking status, alcohol consumption, hypertension, diabetes, alternative healthy eating index and total daily energy intake (continuous, kcal/d)
